# Induction of inflammatory cytokines and toll-like receptors in chickens infected with avian H9N2 influenza virus

**DOI:** 10.1186/1297-9716-42-64

**Published:** 2011-05-18

**Authors:** Nguyen Tai Nang, Joo Sub Lee, Byung Min Song, Young Myong Kang, Hyun Soo Kim, Sang Heui Seo

**Affiliations:** 1 Laboratory of Influenza Research, College of Veterinary Medicine, Chungnam National University, Daejeon, 305-764, Korea; 2 Institute for Influenza Virus: Chungnam National University, Daejeon, 305-764, Korea; 3 Laboratory of Public Health, College of Veterinary Medicine, Chungnam National University, Daejeon, 305-764, Korea

## Abstract

H9N2 influenza virus is endemic in many Asian countries and is regarded as a candidate for the next human pandemic. Knowledge of the induction of inflammatory responses and toll-like receptors (TLRs) in chickens infected with H9N2 is limited. Here, we show that H9N2 induces pro-inflammatory cytokines such as transforming growth factor-beta 3; tumor necrosis factor-alpha; interferon-alpha, -beta, and gamma; and TLR 1, 2, 3, 4, 5, 7, and 15 in trachea, lung, and intestine of infected chickens. In the lung, TLR-15 was dominantly induced. Taken together, it seems that H9N2 infections efficiently induce inflammatory cytokines and TLRs in trachea, lung and intestine of chickens.

## Introduction

Influenza A viruses circulate in aquatic birds such as gulls and shorebirds, and are in evolutional stasis [1]. Some avian influenza viruses are occasionally transmitted to terrestrial birds such as chickens and ducks, and mammals like horses and pigs. Currently, 16 serologically distinct hemagglutinin (HA) and nine neuraminidase (NA) influenza virus subtypes are circulating in aquatic birds [1,2].

H9N2 influenza virus (hereafter referred to as H9N2) was first isolated from diseased chickens in China in 1994. Since then, this virus has been found in many countries including Korea, India, and Pakistan [3-7]. In Eurasia, three distinct H9N2 lineages, Chicken/Beijing/1/94 (Ck/Bei-like), Quail/Hong Kong/G1/97 (G1-like), and Duck/Hong Kong/Y439/97 (Y439-like or Korea-like), are circulating in domestic poultry, with the Ck/Bei-like and G1-like viruses in circulation in China, the Middle East, and Germany [8,9]. Y-439-like viruses were isolated in ducks in Hong Kong in 1997 and have been found in poultry in Korea since 1996 [5,10].

H9N2 can infect humans. In 1999, two cases of mild influenza were reported in two children infected with H9N2 [11] and in 2003 H9N2 also infected a 5-year-old child in Hong Kong [12].

Inflammatory cytokines are suggested to be involved in pathogenesis in humans, pigs, and horses [13-16]. Infections of humans by H5N1 were associated with the expression of MxA protein and interferon-alpha (INF-α) mRNA as demonstrated in autopsy lung tissues [15]. Experimental infection of pigs with swine influenza virus correlated the clinical signs with the levels of INF-α, interleukin (IL)-6, and tumor necrosis factor-alpha (TNF-α) [13]. Infections of horses with equine influenza virus elicited the production of inflammatory cytokines such as INF-α, IL-1β, IL-6, and TNF-α [14].

Toll-like receptors (TLRs) are important for eliciting innate immunity in animals by playing an essential role as pattern recognition receptors that detect infectious pathogens by recognizing the conserved molecular structures known as pathogen associated molecular patterns [17-19]. The recognition of TLRs by pathogens induces the production of reactive oxygen and nitrogen intermediates, and inflammatory cytokines. In chickens, 10 TLR genes (TLR1A, 1B, 2A, 2B, 3, 4, 5, 7, 15, and 21) have been identified [20-23].

The current study was undertaken to investigate the inflammatory cytokines and TLRs induced in H9N2-infected chickens, to further the understanding the early pathogenesis and immune responses in H9N2 infection.

## Materials and methods

### Virus and chickens

The H9N2 representative, A/Chicken/Korea/S21/2004, was grown in 10-day-old fertilized eggs. The experiments were performed in a BSL-3 facility approved by the Korean government. Specific pathogen free (SPF) chickens (White Leghorn) that were 3-4 weeks o1d were purchased from a local farm. Animal work was approved by internal animal ethics committee at Chungnam National University.

### Viral titration

Chickens (*n *= 5 per group) were intranasally (i.n.) infected with 0.5 mL of 10^6 ^log 10 egg infectious dose 50/mL (log_10_EID_50_/mL) of A/Chicken/Korea/S21/2004 and tissues of the trachea, lung, and intestine were collected after chickens were euthanized by cervical dislocation. The collected tissues (0.5 g) were homogenized in 1 mL of phosphate buffered saline (PBS) containing ampicillin and penicillin, and were centrifuged to obtain the supernatant. Virus in the supernatant was determined by log_10_EID_50_/mL as described previously [24]. The viral titer in intestine was the mean of jejunum and colon including rectum.

### Quantification of inflammatory cytokines and TLRs by real-time polymerase chain reaction (PCR)

Total RNA was collected from chickens (*n *= 5 per group) that were infected i.n. with 0.5 mL of 10^6 ^EID_50_/mL of A/Chicken/Korea/S21/2004 using TRIzol reagent (Invitrogen, Carlsbad, CA, USA). Chickens were euthanized by cervical dislocation and tissues samples (0.5 g) of the trachea, lung and intestine were collected in tubes. One milliliter of TRIzol reagent was added to tubes containing tissues and incubated at room temperature for 5 min. Chloroform (200 μL) was added and the solution was mixed by vortexing for 15 s and centrifuged for 15 min, 12 000 rpm, 4°C. The upper RNA-containing band was collected and mixed with 500 μL of isopropanol (Sigma-Aldrich, St. Louis, MO, USA) in a new 1.5 mL tube. Each sample was centrifuged for 10 min, 10 000 rpm, 4°C, and the RNA-containing pellet was washed with 100 μL of 75% ethanol in water by centrifuging for 5 min at 10 000 rpm and 4°C. The washed RNA was resuspended in 50 μL of diethyl pyrocarbonate-treated water.

The mRNAs of chicken inflammatory cytokines and TLRs were quantified using quantitative real-time PCR. To synthesize the cDNA, 1 μL of oligo dT primers (0.5 μmoles) (Promega, Madison, WI, USA) was added to a total volume of 9 μL in a 0.05 mL tube. The mixture was reacted for 5 min at 70°C prior to incubation for 5 min at 4°C. Then, each sample received 4 μL of 25 mM MgCl_2_, 4 μL of 5X reverse transcriptase enzyme buffer, 1 μL of RNase inhibitor, 1 μL of reverse transcriptase, and 1 μL of dNTP (10 mM). Each sample was incubated for 5 min at 25°C, 60 min at 42°C, and 15 min at 70°C. SYBR Green-based real-time PCR was performed using a Roto-Gene 6000 apparatus (Corbett, Mortlake, Australia) and SensiMix Plus SYBR (Quantace, London, UK) based on recommendations of the manufacturer. A duplicate of each sample was run. A total volume of 20 μL containing 2 μL cDNA, 10 μL SYBR mixture, and inflammatory cytokine-specific (Table 1) or TLR-specific primers (Table 2) (1 μL forward primer (20 pmole) and 1 μL of reverse primer (20 pmole)) was used for 40 cycles of PCR: 5 s at 95°C, 15 s at 60°C, and 25 s at 72°C. Cytokine and TLR expression levels in tissues were normalized to those of chicken glyceraldehyde-3-phosphate dehydrogenase (GAPDH). The results of real-time PCR were quantified by the comparative threshold method after deductions of data from uninfected chickens as previously described [25]. The fold change of mRNA of cytokines or TLRs in intestine was the mean of jejunum and colon including rectum.

**Table 1:** Cytokine primer.

Cytokine	Forward Primer	Reverse Primer
TGF-b3	1251F-GGTGGTGAAATCCTGCAAGT	1642R-CCCATTTCCAATCCCTCTTT
TNF-α	57F-CTTCTGAGGCATTTGGAAGC	407R-ACTGGGCGGTCATAGAACAG
INF-α	208F-GACATGGCTCCCACACTACC	556R-AGGCGCTGTAATCGTTGTCT
INF-β	421F-GCTCACCTCAGCATCAACAA	607R-GGGTGTTGAGACGTTTGGAT
INF-γ	320F- TGAGCCAGATTGTTTCGATG	471R-CTTGGCCAGGTCCATGATA
IL-1β	134F-GGATTCTGAGCACACCACAGT	405R-TCTGGTTGATGTCGAAGATGTC
IL-2	83F-TTGGCTGTATTTCGGTAG CA	251R-GTGCACTCCTGGGTCTCAGT
IL-4	99F-GGAGAGCATCCGGATAGTGA	284R-TGACGCATGTTGAGGAAGAG
IL-6	545F-ATCCGGCAGATGGTGATAAA	707R-CCCTCACGGTCTTCTCCATA
IL-8	459F-CATCATGAAGCATTCCATCT	663R-CTTCCA AGGGATCTTCATTT
IL-10	249F-GCTGCGCTTCTACACAGATG	451R-TCCCGTTCTCATCCATCTTC
GAPDH	343F-GACGTGCAGCAGGAACACTA	370R-TCTCCATGGTGGTGA AGACA

**Table 2:** Toll-like receptor primers.

Toll-like receptors	Forward Primer	Reverse Primer
TLR-1	2199F- GCTGTGTCAGCATGAGAGGA	2436R-GTG GTACCTCGCAGGGATAA
TLR-2	421F-GAA AGTTCCCCCTTTTCCAG	666R-AGAGTGCAGAAGGTCCCTGA
TLR-3	1256F-CCTCCTTGGGACACCTGA AA	1494R-ATTCCGCAGTGGATGAAAAG
TLR-4	2624F-GCTGGGCAA AGTGAA AAGAG	2846R-TAAGAACAGCCCGTTCATCC
TLR-5	202F-CCACTGCTGGAGGATTTGTT	416R-TCCAGGATGGAATCTCCA AG
TLR-7	2978F- AGAGACTGGCTTCCAGGACA	3196R- CAGCTGAACATACCGGGACT
TLR-15	893F-CCATCAACAGCCTGGAAACT	1129R-CCTGGTTTCTGACCAAGGAA

### Statistical analysis

Statistical analysis was performed using the Statistical Product and Services Solutions, version 10.0 (SPSS, Cary, NC, USA). ANOVA analysis was performed by comparing infected data to uninfected data. A *P*-value < 0.05 was considered statistically significant.

## Results

### Viral titers in H9N2-infected chickens

**Figure 1:**
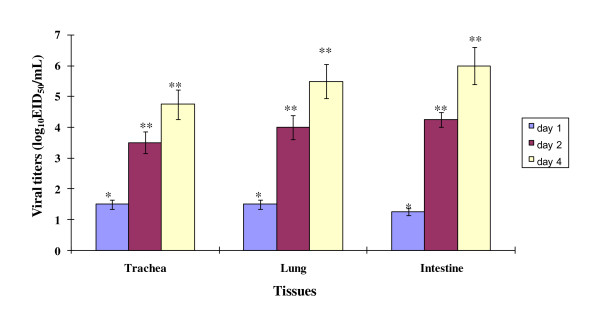
**Viral titers in tissues of H9N2-infected chickens**. Chickens (*n *= 5 per group) were i.n. infected with A/Chicken/Korea/S21/2004 (H9N2) and tissues of trachea, lung, and intestine were collected on 1, 2 or 4 days pi. Tissues of infected or uninfected chickens were homogenized in 1 mL of PBS and the viral titers were determined by log_10_EID_50_/mL. Data represent the mean ± standard errors of the viral titers of five chickens. Statistical analysis was performed by the comparison of data of uninfected chickens. No virus was detected in uninfected chickens. [*P < 0.05], [**P < 0.001].

Chickens (*n *= 5 per group) were exposed to the representative H9N2 to establish that the virus was capable of infection. The viral titers were determined by log_10_EID_50_/mL in tissue samples from trachea, lung, and intestine collected on day 1, 2, and 4 post-infection (pi). H9N2 replicated well in all tissues (Figure 1). The viral titer in tracheas, lungs, and intestine on day 4 pi was 4.75, 5.5, and 6.0 log_10_EID_50_/mL, respectively.

### Inflammatory cytokines in H9N2-infected chickens

**Figure 2:**
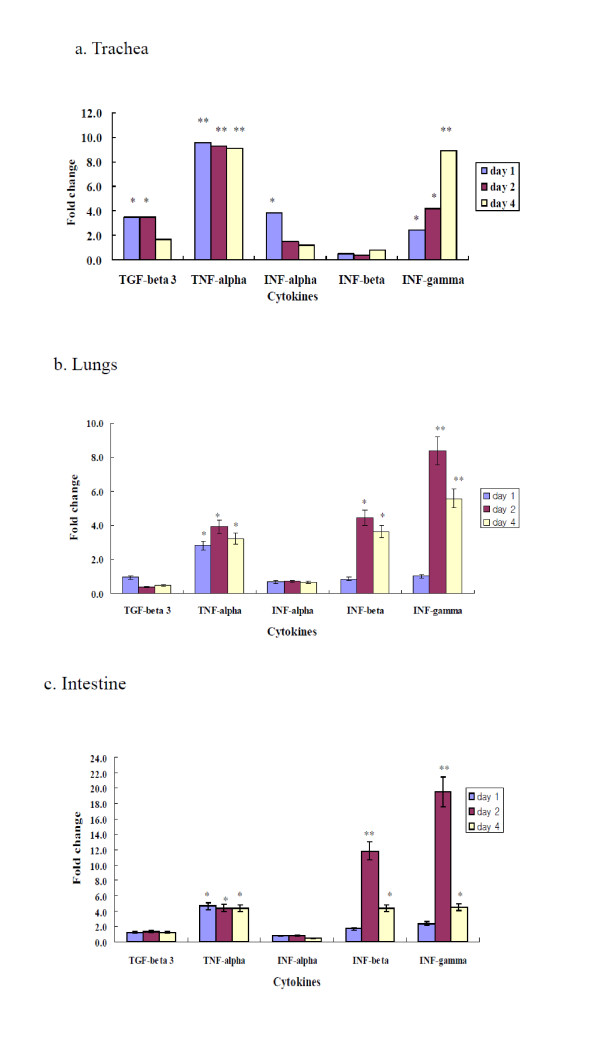
**Inflammatory cytokines in tissues of H9N2-infected chickens**. Total RNA was collected from tissues of infected or uninfected chickens (*n *= 5 per group) with A/Chicken/Korea/S21/2004 (H9N2), and cytokines were quantified using primers specific for chicken cytokines and SYBR Green-based real-time PCR. Data represent the mean ± standard error of five chickens. Panels A, B, and C depict results from trachea, lung, and intestinal tissue. Statistical analysis was performed by the comparison of data of uninfected chickens. [*P < 0.05], [**P < 0.001].

To determine inflammatory cytokines in tissue of trachea, lung, and intestine of H9N2-infected chickens, quantitative real-time PCR was performed using primers specific for chicken cytokines such as TGF-β3, TNF-α, IFN-α, IFN-β, IFN-γ, IL-1β, IL-2, IL-4, IL-6, IL-8, and IL-10, and mRNA of trachea, lung, and intestine of chickens (*n *= 5 per group) on day 1, 2, and 4 pi. In trachea, the inflammatory cytokines (TGF-β3, TNF-α, INF-α, INF-γ) were induced to higher levels in the H9N2-infected chickens than in the uninfected chickens (Figure 2a). In lung, the inflammatory cytokines (TNF-α, INF- β, INF-γ) were induced to higher levels in the H9N2-infected chickens than in the uninfected chickens (Figure 2b). In intestine, the inflammatory cytokines (TNF-α, IFN-β, INF-γ) were induced to higher levels in the H9N2-infected chickens than in the uninfected chickens (Figure 2c). The tissue levels of IL-1β, IL-2, IL-4, IL-6, IL-8, and IL-10 were similar in infected and uninfected chickens (data not shown). In trachea tissue, TNF-α was induced to a greater extent than TGF-β3, TNF-α, INF-α, INF-β, and INF-γ on days 1, 2, and 4 pi. The fold-change of TNF-α in the trachea of chickens on day 4 pi was 9.1, while the fold-change of TGF-β3, INF-α, INF-β, and INF-γ was 1.6, 1.2, 0.8, and 8.9, respectively (Figure 2a). In lung tissue, INF-γ was induced higher in the infected chickens than TGF-β3, TNF-α, INF-α, and INF-β on days 2 or 4 pi. The fold-change of INF-γ in the lungs in infected chickens was 5.6, while that of TGF-β3, TNF-α, IFN-α, and INF-β was 0.5, 3.2, 0.7, and 3.6 on day 4 pi, respectively (Figure 2b). In intestine tissue, INF-γ was induced more in the infected chickens than TGF-β3, TNF-α, INF-α, and INF-β. The fold-change of INF-γ in intestine tissue was 19.3 on day 2 pi, whereas that of TGF-β3, TNF-α, INF-α, and INF-β was 1.3, 4.5, 0.8, and 11.7 on day 2 pi, respectively (Figure 2c).

### Induction of TLRs in H9N2-infected chickens

**Figure 3:**
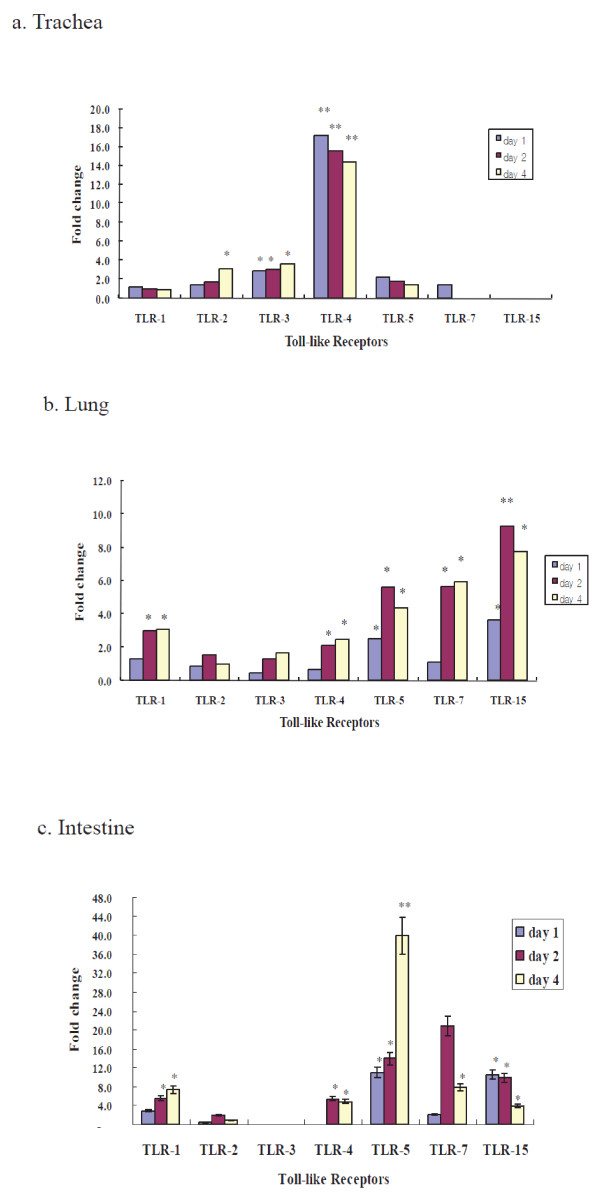
**TLRs in tissues of H9N2-infected chickens**. Induction of TLRs using total RNA (Figure 2) was quantified using primers specific for chicken TLRs and SYBR Green-based real-time polymerase chain reaction. Data represent the mean ± standard error of five chickens. Panels A, B, and C depict results from trachea, lung, and intestinal tissue. Statistical analysis was performed by the comparison of data of uninfected chickens. [*P < 0.05], [**P < 0.001].

We determined the induction of TLRs in trachea, lung, and intestine tissue in those chickens examined in the experiment summarized in Figure 2, since TLRs are involved in the induction of inflammatory cytokines. In trachea tissue, among the tested TLRs, TLR-15 was not induced in the H9N2-infected chickens, TLR-1 was less induced in the H9N2-infected chickens, and TLR-4 was the highest induced (17.1-fold change on day 1 pi) (Figure 3a). In lung tissue, all tested TLRs were induced in the H9N2-infected chickens with TLR-15 was the highest induced (9.2-fold change on day 2 pi) (Figure 3b). In intestine tissue, TLR-3 was not induced and TLR-5 was the highest induced, with a 39.2-fold change on day 4 pi (Figure 3c).

## Discussion

H9N2 is endemic in many countries including Korea and China, and is regarded as one of the candidates for the next human pandemic. We studied mRNA expression of inflammatory cytokine and TLRs in H9N2-infected chickens to understand the early inflammatory responses and pathogenesis of the infection. The data demonstrate that H9N2 infects trachea, lung, and intestine tissue, and induces the production of inflammatory cytokines and TLRs in these tissues.

Our data showed that cytokine inductions increased as viral titers increased in tracheas and lungs of chickens infected with H9N2. In tracheas, the higher inductions of TNF-α and INF-γ were made on 4 days pi when viral titer peaked. In lungs, TNF-α, INF-β, and INF-γ were induced higher on 4 days when viral titer peaked. Previous studies suggest that cytokines may be involved in both clearance of virus and pathological tissue damage. A study on a chicken macrophage cell line infected with H9N2 showed that inflammatory cytokines such as IL-1β and IL-8 were up-regulated [26]. However, this study did not measure the induction of inflammatory cytokines in vivo. In vivo studies in pigs or horses infected with influenza virus indicated that the clinical signs are correlated with the production of inflammatory cytokines [13,14]. When 3-week-old piglets were infected with swine H1N1 influenza virus (A/Swine/Belgium/1/98), inflammatory cytokines such as INF-α, IL-6, IL-1, and TNF-α peaked in broncho-alveolar lavage fluid 24-39 h pi, when viral titers and clinical signs of infected pigs were the highest [13]. In horses infected with H3N8 equine influenza virus (A/Equine/Kildare/89), inflammatory cytokines such as INF-α, IL-1β, IL-6, and TNF-α were up-regulated when the amount of inflammatory cytokines were determined by quantitative real-time PCR [14]. It was also reported that the infections of humans with highly pathogenic (HP) H5N1 influenza virus and the infection of mice with HP H5N1 or 1918 pandemic H1N1 influenza virus could elicit the production of inflammatory cytokines [15,27]. The autopsy lung tissues from human patients infected with HP H5N1 influenza virus showed the elevated expression of pro-inflammatory MxA, IFN-α, and IP-10 compared to control lung tissues of humans [15]. Infection of mice with HP H5N1 or pandemic 1918 H1N1 influenza virus led to the rapid infiltration of macrophages and neutrophils into the lungs, resulting in the acute inflammation with the production of inflammatory cytokines such as IL-1α, IL-6 and INF-γ [27].

When we determined the induction of TLRs in trachea, lung, and intestine tissue, TLR-4, TLR-15, and TLR-5 were dominantly induced in trachea, lung, and intestine, respectively. The results suggest that the different tissues can respond to H9N2 using the different TLRs and that tissue specific expression of TLRs can be induced. TLR-15 was induced higher in lungs, but it was not induced in tracheas of chickens infected with H9N2 influenza virus. Targeting theses TLRs for therapeutic purpose can be one of ways to defend chickens from the infections of avian influenza viruses. The recent study showed that the pre-stimulation of TLR-2 and TLR-4 by their ligand could increase the resistance to highly pathogenic H5N1 influenza viruses in mice [28]. Information on TLR induction in chickens infected with influenza viruses is scarce. A previous study with a chicken macrophage cell line infected with H9N2 showed that TLR-7 expression was up-regulated and was involved in cytokine responses [26]. When chickens were infected with Marek's disease virus via the respiratory route, the expression of TLR-3 and TLR-7 increased in the lungs [29].

When we determined the kinetics gene expression of inflammatory cytokines or TLRs some genes increased as time elapsed, but others did not. In trachea, the induction of TNF-α was similar on 1, 2 or 4 days pi, while the induction of INF-γ increased as time lapsed. In trachea, TLR-4 was induced highest on 1 day pi, rather than 4 days pi, while in intestine, the induction of TLR-5 was induced highest on 4 days pi rather than 1 day pi.

In conclusion, infection of chickens with H9N2 may induce the production of inflammatory cytokines in trachea, lung, and intestinal cells by inducing different TLRs.

## Competing interests

The authors declare that they have no competing interests.

## Authors' contributions

NTN and JSL carried out the experiment on cytokines and toll-like receptors in the infected chickens and drafted the manuscript. BMS and YMK helped the animal experiment. HSK preformed the statistical analysis. SHS conceived the study, and participated in its design and coordination. All authors have read and approved the final manuscript. All authors read and approved the final manuscript.
